# Adherence to the Mediterranean Diet Across the League of Arab States: A Systematic Review

**DOI:** 10.3390/healthcare13172217

**Published:** 2025-09-04

**Authors:** MoezAlIslam E. Faris, Nada Benajiba, Basil H. Aboul-Enein, Katia Abu Shihab, Rasha Alshaalan, Rehab Aldahash, Fatmah Almoayad

**Affiliations:** 1 Department of Clinical Nutrition and Dietetics, Faculty of Allied Medical Sciences, Applied Science Private University, Amman 11931, Jordan; 2 Joint Research Unit in Nutrition and Food RDC-Nutrition AFRA/IAEA, Ibn Tofail University-CNESTEN, Kenitra 14000, Morocco; benajibanada@gmail.com; 3 Health & Society Program, College of Arts & Sciences, University of Massachusetts Dartmouth, 285 Old Westport Rd, North Dartmouth, MA 02747, USA; baboulenein@umassd.edu; 4 London School of Hygiene & Tropical Medicine, Faculty of Public Health and Policy, 15-17 Tavistock Place, London WC1H 9SH, UK; 5 Research Institute of Medical and Health Science (RIMHS), University of Sharjah, Sharjah P.O. Box 27272, United Arab Emirates; katia.hazim@outlook.com; 6 Department of Health Sciences, College of Health and Rehabilitation Sciences, Princess Nourah Bint Abdulrahman University, P.O. Box 84428, Riyadh 11671, Saudi Arabia or rashaalshaalan@gmail.com (R.A.); raaldahash@pnu.edu.sa (R.A.); faalmoayad@pnu.edu.sa (F.A.)

**Keywords:** sustainable diet, religion and health, diet history, MENA region, dietary adherence, geographic variation

## Abstract

**Purpose:** The Mediterranean diet (MD) is associated with significant health benefits. However, adherence varies considerably, influenced by sociocultural and geographical factors. This review was designed to synthesize existing evidence on the prevalence of MD adherence in different Arab countries and identify sociodemographic, cultural, and behavioral factors associated with adherence. **Methodology:** Sixteen databases were searched to identify relevant articles, using MeSH search terms related to MD and its applicable terms, adherence, and the names of the 22 Arab countries. **Findings:** Out of approximately 2400 articles searched, nine articles were selected, investigating adherence to the MD across Arab League countries and exploring the impact of geographic location on dietary practices. Examined Arab populations showed generally moderate adherence to the MD. Wide variability was observed in adherence levels among the different Arab countries. This variability arises from a complex interplay of factors, including access to specific foods, economic considerations, cultural traditions, and the influence of globalization on dietary habits. Our review highlights the role of these factors in contributing to the observed heterogeneity in MD adherence across the Arab League, examining the prevalence of various MD assessment tools and their respective strengths and limitations within this specific context. **Conclusions:** The findings underscore the need for culturally sensitive and geographically tailored strategies that enhance adherence to the MD’s protective effects across all countries in the Arab League.

## 1. Introduction

The Mediterranean diet (MD) has been recognized as a health-improving, disease-preventing, and effective anti-obesogenic diet pattern for improving public health and quality of life, as well as decreasing the incidence of various non-communicable diseases [1,2,3]. It is commonly followed in countries bordering the Mediterranean basin [4,5,6]. The MD comprises fruits, vegetables, whole grains, legumes, fish, olive oil, nuts, and small amounts of low-fat dairy products. Evidence-based science suggests that the MD reduces the incidence and prevents the progression of overweight and obesity [7]; metabolic syndrome [8]; diabetes and its complications, such as kidney disease, especially T2D [9,10,11]; cardiovascular diseases [12,13]; cancers [14]; obesity-related morbidities [9,15]; depression [16]; and cognitive decline [12,17], improving cognition in older adults [18] and lowering stress hormone levels [19]. Following this diet can decrease overall mortality by 9%, and a 2-point increase in adherence score can lead to a 25% reduction in mortality from all causes [20,21]. A subgroup analysis revealed that the MD has a stronger inverse association with all-cause mortality in participants residing in the Mediterranean region compared to those in non-Mediterranean areas, making it particularly beneficial for individuals living in countries where the traditional MD is commonly consumed [22]. This health-improving, disease-preventing effect is attributed to the presence of a multitude of bioactive substances with various beneficial effects, including antioxidant, anti-inflammatory, anti-carcinogenic, and anti-thrombotic properties [23].

Given the health significance of adherence to the MD, numerous studies have repeatedly examined this behavior across various Mediterranean and non-Mediterranean countries. A comprehensive systematic review of Mediterranean countries analyzed 50 studies, including 17 from Spain, 10 from France, nine from Italy, six from Greece, and eight from other Mediterranean countries. The participant numbers in these studies ranged from approximately 1000 to over 94,000. While most research focused on European Mediterranean nations, fewer studies explored adherence in the Middle East and North Africa. Overall, the findings of that review indicated that the majority of populations exhibited low-to-moderate adherence to the MD, as reflected by both mean adherence scores and the prevalence of low or moderate adherence categories [6]. For non-Mediterranean countries such as Asia, North America, and Australia, the adherence, implementation of MD interventions, barriers, and facilitators to adhere to the MD were examined in several studies [24,25,26,27,28,29]. Conversely, there is a scarcity of studies investigating adherence to and knowledge of the MD in Central and South America, among Caribbean countries, as well as African countries [25].

The Arab League is a regional political and economic union comprising 22 Arab states that span North Africa and West Asia, thus including both Mediterranean and non-Mediterranean countries. The Arab League encompasses a vast geographical area, spanning over 13 million square kilometers, including a diverse population of approximately 473 million people. This vast region borders several bodies of water, including the Mediterranean Sea. While Arabic serves as the lingua franca, the area is ethnically diverse. As part of the Arab League, the Eastern Mediterranean countries, which include Egypt, Lebanon, Syria, Jordan, and the occupied Palestinian territories, play a significant role in the region’s politics, economy, and demographics. Despite the challenges faced by the area, including political instability, economic disparities, and social issues, the Arab League continues to play a crucial role in regional affairs, addressing topics such as security, economic cooperation, and cultural exchange [30].

In the Arab world, cardiovascular disease (CVD) was found to be the leading cause of death. The incidence rate of major CVD was 12.7 per 1000 person-years [31]. Further, the number of cancer cases in Arab countries is on the rise. It is projected to increase by 1.8 times by the year 2030. In countries like Saudi Arabia and the United Arab Emirates, cancer is one of the top three causes of death. Furthermore, cancer is the second leading cause of death in North African countries [32]. In the Middle East and North Africa region, it is estimated that one in ten people has diabetes. Furthermore, six out of the top ten countries with a high estimated prevalence of diabetes were Arab nations [33]. The high morbidity and mortality rates underscore the critical role of the MD in mitigating chronic diseases in the Arab region. The decline in adherence to the MD among Mediterranean countries in recent years has led to an increase in non-communicable diseases, with many individuals adopting a Western-style dietary pattern [1]. Studies have shown that the general population in Mediterranean countries exhibits low-to-moderate adherence to the MD. Despite moderate adherence to the MD among countries bordering the Mediterranean Sea over the past decade, some variation exists within and between countries [2,6]. The MD can vary based on cultural and socioeconomic factors across different Mediterranean countries [34].

Given the rising prevalence of chronic diseases such as CVD, T2D, and obesity in the Arab world, and considering the potential health benefits of the MD, a systematic review focusing on adherence to this dietary pattern across the League of Arab States is crucial. Therefore, this review was designed to synthesize existing evidence on the prevalence of MD adherence in different Arab countries and identify sociodemographic, cultural, and behavioral factors associated with adherence. By understanding the factors influencing adherence to the MD in Arab populations, policymakers can develop targeted interventions to promote healthy eating habits and reduce the burden of chronic diseases. The results will also provide valuable information for healthcare professionals to counsel patients on the benefits and practical aspects of adopting a Mediterranean-style diet. This systematic review will significantly contribute to our understanding of the MD in the Arab context and inform future research and public health initiatives. While a few studies discuss adherence to the MD in specific Arab countries, no systematic review has attempted to examine adherence across the League of Arab States.

## 2. Methods

A systematic review of studies reporting on adherence to the MD among adults from Arab League countries was conducted according to PRISMA 2020 guidelines [35].

### 2.1. Literature Search

For this review, Arabic-speaking countries were defined as the 22 member countries of the League of Arab States [36,37]. To conduct a comprehensive literature search, sixteen databases were searched up to and including November 2024. The search strategy employed a combination of terms, keywords, and search phrases tailored to each respective database. Rayyan QCRI software (2024) was used to assist in the screening process and to select the studies. Electronic databases used, along with relevant search periods and terms, are listed in Supplementary Table S1. After removing duplicates (performed by MEF, BAE, and FA), articles were screened against the inclusion and exclusion criteria first by title and then by abstract (MEF, RAA, and FA). Excluded articles were then confirmed by a second author (NB), and discrepancies were resolved by discussion and confirmed with another author (ED). The full texts were screened for final selection by MEF, BAE, FAA, and RAA.

### 2.2. Selection Criteria for Studies

Studies were included if they targeted adults (at least 18 years old); were conducted in 1 or more of the 22 states of the League of Arab; used a dietary assessment scoring tool to quantify adherence to the MD such as Mediterranean diet Adherence Screener (MEDAS), Mediterranean diet Scale (MDS), MedDietScore, and others [38,39,40]; and reported either a mean or median adherence score, or a distribution of adherence categories (e.g., low, moderate, high) within the general population and/or relevant subgroups based on age or sex. Studies that focused solely on populations with chronic illnesses, co-morbidities, or a high risk of nutrition-related disorders (such as inflammatory bowel disease, cardiovascular diseases, diabetes, kidney diseases, or wasting conditions like cancer and HIV), or those with conditions impairing independent food choice (e.g., documented dementia, Alzheimer’s disease, psychological disorders such as schizophrenia) were excluded. Additionally, studies involving specific subpopulations such as pregnant women, older adults, or athletes were excluded to maintain focus on the general population. We also excluded studies with a sample size of fewer than approximately 1000 participants to ensure representativeness, as well as studies assessing MD adherence amid the COVID-19 pandemic and lockdown periods, since these do not reflect typical and habitual dietary and lifestyle behaviors. Inclusion and exclusion criteria are listed in Table 1.

### 2.3. Data Extraction and Quality Assessment

Data from each included article were extracted and organized into a table within an Excel file by two authors (BAE and KA). The following data were extracted from the articles and tabulated: author name and year of publication, target population/country, study design, the dietary intake assessment tool, and the score used to assess MD adherence. Disagreements were resolved through consensus with the assistance of a third reviewer (MEF). Additionally, the mean MD adherence score and/or the distribution across adherence categories in the general population were extracted, and any assessments of sociodemographic variables as determinants of MD adherence were recorded. Moreover, if a study targeted multiple countries, only the results of the Arab countries were included in the current review.

The methodological quality of included studies was assessed using the Joanna Briggs Institute (JBI) Critical Appraisal Checklist for Analytical Cross-Sectional Studies [41]. This tool evaluates key domains, including the clarity of inclusion criteria, the validity and reliability of exposure and outcome measurements, the identification and adjustment for confounding factors, and the appropriateness of statistical analysis. Each item was rated as “Yes,” “No,” “Unclear,” or “Not Applicable,” with overall risk of bias categorized as low, moderate, or high based on the proportion of criteria met. KA and MEF performed the quality assessment, and disagreements were discussed until a consensus was reached.

### 2.4. Data Analysis

For data analysis, the mean MD adherence scores reported in the included studies were translated into qualitative categories—high, moderate, or low adherence—using the respective researchers’ thresholds and standardized statistical tertiles to facilitate systematic comparison.

## 3. Results

### 3.1. Study Selection

**Figure 1:**
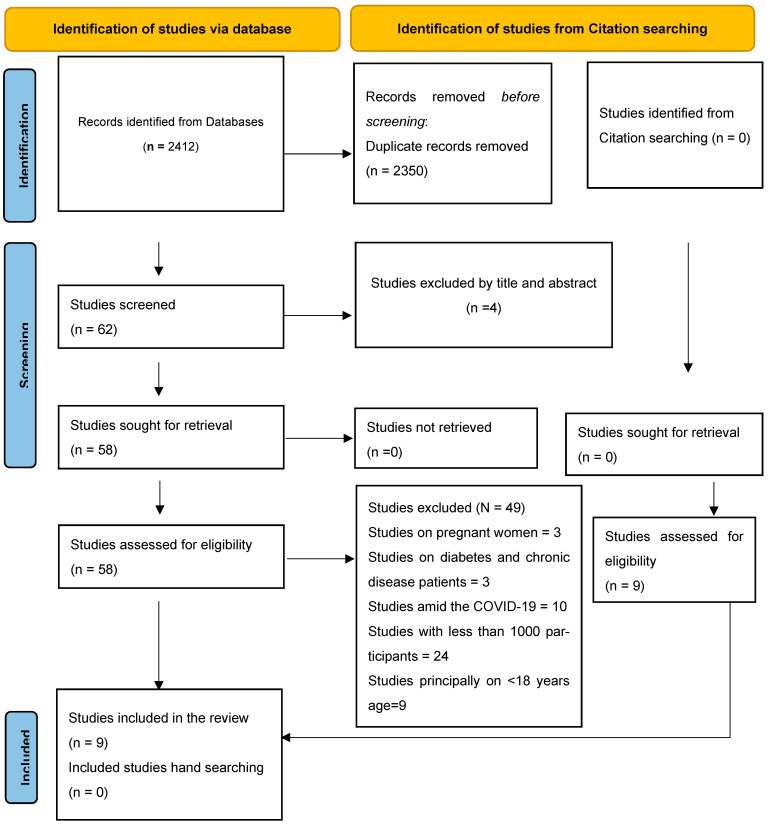
PRISMA figure for screening process flow.

The systematic review process began with the identification of 2412 records from databases, followed by the removal of 2350 duplicate records. After screening the remaining 62 studies, four were excluded based on their titles and abstracts. All 58 remaining studies were retrieved and assessed for eligibility, with 49 being excluded due to focusing on pregnant women (3), diabetes and chronic disease patients (3), COVID-19 (10), having much fewer than approximately 1000 participants (25), or primarily focusing on individuals under 18 years of age (8). Additionally, nine studies from an independent search were assessed for eligibility, and none were excluded from the analysis. Ultimately, the nine studies were included in the review, with no additional studies identified through hand-searching (Figure 1).

### 3.2. Study Characteristics

The basic characteristics of the selected articles are extracted and tabulated in Table 2. The nine included research articles were conducted between 2012 and 2024, with an increasing trend towards recent years, as two publications [42,43] were conducted in 2021 and four [44,45,46,47] were conducted in 2024. Research on MD adherence of large participant samples has been conducted in only seven countries out of the 22 Arab League countries. The studies spanned various countries, including three in Lebanon [46,48,49], four in Morocco [42,47,50], including one in joint with Tunisia [45], and two in the Gulf, namely Saudi Arabia, Oman, and Kuwait [43], and the UAE [44]. Moreover, some studies were conducted simultaneously in more than one country for comparative purposes, such as the study by Biggi et al. in Tunisia and Morocco [45] and the study by Shatwan et al., conducted in three Gulf countries (Saudi Arabia, Oman, and Kuwait) [43].

All studies adopted the cross-sectional design, providing a snapshot of dietary habits and health outcomes at a single point in time; however, some studies have followed a descriptive approach towards the prevalence of MD adherence and its determinants [44,45,49,50], while others have analyzed the Mediterranean adherence’s association with other outcomes [42,43,46,47,48]. The outcomes included environmental footprints [48], overweight and obesity risk [42,43,47], and cardiovascular disease risk factors [46]. Adherence to MD assessment tools varied between the different studies; the most predominant tool being used was the Mediterranean Diet Adherence Screener (MEDAS) from the Spanish cohort study (PREDIMED), used by three articles [43,44,45], followed by the Mediterranean Diet Quality Index (Med-DQI) used by two [48,49]. Table 3 provides an overview of the MD score systems used in the included studies.

### 3.3. MD Adherence Levels

Table 4 summarizes MD adherence findings, including the reported mean adherence score, its classification based on both study-specific and standardized tertile cut-offs, and the distribution of participants across adherence levels. Seven out of the nine studies have reported the mean score of MD adherence for their assessed participants. Based on each study’s own utilized classification threshold, the overall means of two studies were classified as low, two as moderate, and only one as high adherence. The other two studies have not mentioned using cut-off ranges. Conversely, when categorizing the reported means using statistical tertiles, six studies’ means fall into the moderate category, and one falls into the low category of MD adherence. These findings align with the reported distributions of MD adherence among studies that provided this data: four studies showed a predominant proportion of participants with moderate adherence, one study reported a majority with high adherence, and one study indicated a majority with low adherence.

The environmental impact was presented by Naja et al. in terms of water use, energy use, and greenhouse gas as environmental footprints of MD adherence [48]. Meanwhile, the impact of MD adherence on metabolic health was studied specifically as an obesity risk and as body anthropometrics [42,43] and cardiovascular disease risk factors [46].

### 3.4. MD Adherence Subgroups

Only six out of the nine included studies have analyzed the association between MD adherence and different sociodemographic groups and lifestyle factors [42,43,44,46,47,50]. Across the included studies, sociodemographic factors such as biological sex, educational level, monthly income, residency, housing, age group, marital status, and lifestyle variables, including physical activity, smoking status, and BMI, were the most frequently assessed variables in their association with MD adherence. Married individuals [44,50], older age groups [44,46], and women [43,46] were the most common sociodemographic groups to exhibit significantly higher adherence than their counterparts.

### 3.5. Quality Assessment of the Included Studies

The included studies underwent quality assessment via the JBI critical appraisal tools for analytical cross-sectional study designs [41]. Most studies met the key domains of the JBI critical appraisal tool, including clear inclusion criteria, detailed descriptions of study populations and settings, objective measurement of the condition, and valid outcome assessment methods. However, the reliability of exposure measurement was unclear in a few studies, which did not specify how questionnaires were administered or whether data collectors received training. Reporting on confounding factors, including their identification, handling strategies, and appropriateness of adjustment, was also inconsistent, and in some cases did not align with JBI criteria. Based on these domains, seven studies were assessed as having a low risk of bias, while two were classified as moderate risk. Detailed scores for each domain are provided in Supplementary Table S2.

## 4. Discussion

This systematic review provided an overview of MD adherence among the general population living in Arab League countries, including individuals who traditionally follow the MD, such as those in the Levant countries. The search strategy and application of strict inclusion/exclusion criteria identified nine articles, mostly reporting moderate levels of adherence to the MD. The moderate adherence may be attributable to the gradual decline of traditional dietary practices due to urbanization and globalization experienced worldwide and within the Arab region [59]. Nonetheless, it remains unclear whether these findings truly reflect dietary behavior or whether limitations instead influence them in the cultural suitability of the MD adherence tools used in Arab populations. Among the assessed studies, only four studies (Naja et al. [48,49], Zeenny et al. [46], and Kinany et al. [42]) developed and applied culturally relevant MD assessment tools. In contrast, most other studies relied on more widely used adherence tools, such as MEDAS [51], MDS [52,53], or MedDietScore [54], which were originally developed and validated in Greece and Spain. This reliance may introduce discrepancies, as the scoring criteria of non-native tools may not fully align with the traditional Mediterranean dietary practices of Arab populations, calling into question their contextual validity and cultural appropriateness. In general, the absence of a univocally accepted tool to assess MD adherence, combined with the abundance of developed methods, remains a persistent methodological limitation in this field [40].

Additionally, our findings have assessed the adherence to the MD in large population samples in only 7 of the 22 Arab League countries, with most studies being concentrated in Lebanon and Morocco. This may reflect regional differences in research attention, likely influenced by the proximity of these countries to the Mediterranean basin and the alignment of MD principles with their native culinary traditions. As such, further research is essential in other member states to create a comprehensive understanding of dietary adherence across the region, and to evaluate the MD’s role and adherence in their population, particularly in non-Mediterranean Arab countries like the Gulf. Investigating the cultural, economic, and social contexts of food practices can aid in tailoring interventions that resonate more effectively with local populations [60], given that the existing versions of the MD are largely shaped by their historical and cultural roots from their originating region [61,62,63].

Comparing findings of the current work with previous research on MD adherence shows that adherence among the tested Arab populations is consistent with adherence among populations in European and non-European Mediterranean countries [6], which showed low-to-moderate adherence. Interestingly, a slightly higher adherence was reported among the examined Arab countries, which had a higher prevalence of moderate adherence. Furthermore, our findings align with those of a systematic review assessing adherence to the MD among the global population. Examining fifty-seven studies with more than one million apparently healthy adults from Europe, the United States, Asia, Australia, and Africa, the study found that adherence to the MD was moderate [64].

A historical perspective on the Arab world’s influence is crucial for understanding the findings of this review on the MD. The historical perspective reveals a significant relationship between Arab culture and the evolution of the MD [63]. The ninth-century arrival of Arabs in Southern Italy marked a pivotal moment, enriching existing dietary patterns with new ingredients, culinary techniques, and food preparation methods. They introduced a wealth of previously unknown ingredients, including dried pasta, rice, various spices (such as cinnamon, cloves, and nutmeg), citrus fruits (such as lemons and oranges), and sugar cane, significantly diversifying the diet beyond the traditional “bread–olive oil–wine” triad [63].

Beyond introducing new ingredients, the Arabs brought a new culinary imagination, developing innovative ways to prepare food. Dried pasta became a staple due to its practicality, and the introduction of sugar cane spurred a rise in confections and sweets, altering traditional dessert consumption. Unlike the previously existing Greco-Roman and “barbarian” dietary models, which were either vegetarian or focused on meat and dairy, the Arab influence shifted the focus toward carbohydrates, with rice and pasta becoming essential components of the diet. Their expertise in preserving food, such as drying pasta, expanded options and impacted long-term food availability. The Arab contribution, therefore, is not merely additive but transformative, resulting in a more diverse, complex, and carbohydrate-rich dietary pattern that has become a significant aspect of the modern MD; thus, their influence is crucial to recognize within any discussion of the diet’s historical development [63].

Moving back, archeological evidence from Tell Tweini’s (an archeological site located 1 km east of the modern city of Jableh, Syria) Middle Bronze Age reveals a diet remarkably similar to the contemporary MD, including wheat, olives, pulses, dairy products, and small amounts of meat. This aligns with isotopic data from the Eastern Mediterranean, suggesting the “modern” MD has ancient roots in the region, dating back to at least the Bronze Age [65].

Olive oil’s centrality in the MD is deeply rooted in its nutritional value, culinary versatility, and historical significance across the Mediterranean basin [66], particularly within the Arab world. While not explicitly mentioned in the Holy Quran, Islamic traditions often associate olive oil with purity and well-being, fostering a cultural preference for its use [67]. However, religious affiliation alone does not fully explain adherence to this core MD component; economic factors, food availability, and evolving dietary trends also significantly impact consumption [68]. The relationship between religious belief and olive oil use within Arab communities is complex, varying across regions and influenced by the interplay of cultural practices, historical ties, economic realities, and the impact of globalization on dietary habits [69]. Therefore, while cultural preferences for olive oil are strong in many Arab communities, these are often interconnected with broader cultural and social factors rather than solely driven by religious observance.

Only seven studies in our review have reported adherence to MD across different sociodemographic and lifestyle factors. Among those that did, the most common findings were that women, older adults, and married individuals tended to exhibit significantly higher adherence to the MD compared to their respective counterparts. These results align with behavioral nutrition research suggesting that women and older populations often report greater health consciousness and dietary regulation [70,71]. Meanwhile, the association between marital status and MD adherence may reflect shared household norms or increased social support for healthy eating [72,73]. Future research should prioritize the standardized reporting of sociodemographic and lifestyle factors to elucidate their role in shaping MD adherence and to inform targeted public health interventions.

Our review also notes a growing interest in the MD within the scientific community, where the evaluation of MD adherence across large population samples has been particularly conducted in recent years. This shift may indicate a greater recognition of the MD’s health benefits, in the context of the rising rates of non-communicable diseases linked to dietary habits [74,75], such as obesity, in Arab countries. Several studies have shown that obesity is prevalent among female participants in Arabic-speaking countries, especially in Morocco and the Occupied Palestinian Territories [1]. Moreover, the low MD adherence rates, like in the Gulf, found in the current review, may signify the reported increase in the risk of diet-related health issues [76], emphasizing the need for targeted interventions, educational programs, and public health campaigns that encourage adherence to MD principles. For instance, low levels of adherence to the MD among adults from the Gulf countries, Saudi Arabia, Oman, and Kuwait, are found to be associated with increased obesity indicators, hip circumference, and body mass index. The Gulf countries have a high incidence of overweight and obesity, with 60% and 30% of the population affected, respectively [43]. Kuwait has the highest obesity rate in the region, followed by Saudi Arabia, while Oman boasts the lowest rate [43]. Given such prevalences, it becomes crucial to evaluate the impact of MD adherence on health-related outcomes in this region.

Four articles in our review studied the effects of MD adherence on health-related outcomes [42,43,46,47], where MD adherence has shown significant associations with favorable health indicators, such as a lower BMI and hip circumference [43,47]. Nonetheless, more similar research linking MD adherence to health parameters like obesity risk and chronic diseases in large participant samples is needed to promote the MD as part of broader health strategies effectively [7,77], which include targeted interventions, educational programs, and public health campaigns that encourage adherence to MD principles [60].

Finally, while the majority of studies were rated as having a low risk of bias based on the JBI critical appraisal tool, suggesting generally good methodological quality, the findings also highlight a need for more transparent and consistent reporting of data collection procedures and confounding adjustment in future research. Several gaps remain in the literature, suggesting pathways for future research. Longitudinal studies are needed to track changes in MD adherence amid global challenges such as the COVID-19 pandemic, as such disruptions can significantly impact eating behaviors and health outcomes. Additionally, exploring the environmental and economic aspects of dietary adherence could shed light on the broader implications of food choices within the region. Furthermore, the relationship between adherence to the MD and various socio-demographic variables, health outcomes, and lifestyle factors warrants further exploration. Lastly, expanding the scope of the target countries, including designing public campaigns related to the correlation of MD adherence with health risk reduction, is recommended for future research.

## 5. Conclusions

This is the first paper to systematically review the scientific literature pertaining to MD adherence, specifically among adults residing in Arab League countries. Arab populations have generally shown moderate adherence to the MD over the last two decades. Still, there is a need to improve adherence to the MD among younger and older adults, as well as among both men and women, in countries of its origin. This requires the implementation of appropriate health promotion and nutritional policies and interventions. Health promotion efforts aimed at enhancing adherence to medical advice can have a substantial impact on a broad range of health outcomes for residents. In summary, while this systematic review highlights the progress made in researching MD adherence in the Arab League, it emphasizes the need for concerted efforts to address regional disparities and promote adherence through public health initiatives. By fostering a deeper understanding of the factors influencing dietary habits and promoting adherence to healthy eating patterns, the potential to improve overall health outcomes in the region is substantial. As the body of research continues to grow, collaboration among countries within the Arab League will be vital in creating a unified approach to nutrition and health promotion.

## Figures and Tables

**Table 1:** Criteria for inclusion and exclusion of studies.

Parameter	Inclusion Criteria	Exclusion Criteria
**Date Range**	2010 to November 2024	N/A
**Population**	At least around 1000 participants in the sample size. Arabic-speaking populations that reside in an Arab League member state.	Much less than 1000 participants as the sample size. Non-Arab or Arabic-speaking populations. Arab diaspora residing outside an Arab League member state. Individuals with chronic illnesses or co-morbidities (e.g., IBD, CVD, diabetes, kidney disease, cancer, HIV), or with impaired food autonomy (e.g., dementia, Alzheimer’s, schizophrenia). Specific subpopulations (e.g., pregnant women, centenarians or elderly people, athletes). Studies conducted amid the COVID-19 pandemic.
**Measures of interest**	Mediterranean diet adherence	N/A
**Language**	English	All other languages.
**Study type**	Peer-reviewed original research articles	Non-peer-reviewed articles. Study protocols. Narratives. Similar article types. Gray literature. Communications. White papers. Conference proceedings.

**Abbreviations:** N/A—Not applicable.

**Table 2:** Characteristics of the included studies and summarized results.

Authors, Year	Target Population (Country)	Study Design	Sample Size *	MD Assessment Tool or Scoring System	Results
Naja et al., 2019 [48]	Adults over 20 years old (Lebanon)	Cross-sectional	2610	LMD, rMED score, Med-DQI, aMed	Low adherence to the MD was observed, with the majority of participants falling between low and moderate adherence, and a minority having high adherence. A higher adherence to the MD was associated with lower water use. All four MD scores were associated with lower greenhouse emissions.
El Kinany et al., 2021 [42]	Adults over 18 years old (Morocco)	Cross-sectional	1492	MMD	Close adherence to the MD was associated with reduced overweight/obesity risk among Moroccan adults. Those with high adherence to the MD had a 39% reduced risk of excess weight compared with participants in the lowest compliance category.
Shatwan et al., 2021 [43]	Adults 20–55 years old (Gulf countries: Saudi Arabia, Oman, Kuwait)	Cross-sectional	961	MEDAS	The highest adherence to the MD was associated with a decrease in two obesity indicators, body mass index and hip circumference. Low adherence to the MD was reported among participants from three Gulf countries.
Naja et al., 2015 [49]	Adults between 20 and 55 years old (Lebanon)	Cross-sectional	2048	MedDietScore, IMI, rMED score, Med-DQI, MDS, Derivation of the LMD	Men, smokers, younger and less physically active participants, and those with lower education levels were less likely to adhere to an MD dietary pattern.
El Rhazi et al., 2012 [50]	Adults over 18 years old (Morocco)	Cross-sectional	2214	A simplified score of MDS	Adherence to the MD is declining across the Moroccan population, regardless of age or education level. This is especially pronounced in rural communities, among individuals who live alone, and among those residing in affluent households.
Elmskini et al., 2024 [47]	Adults over 18 years old (Morocco)	Cross-sectional	1776	MedDietScore	Compared to students of medical and paramedical sciences, those in both human and social sciences, natural sciences, and engineering showed higher MD adherence scores. Participants who attended nutrition-related training had higher MD adherence scores than those who did not. Similarly, non-overweight students had significantly higher MD adherence scores compared to their overweight/obese peers.
Hashim et al., 2024 [44]	Adults over 25 years old (UAE)	Cross-sectional	1314	MEDAS	The study participants had a moderate adherence score. The MD adherence score was associated with physical activity. Nutrition information from dietitians and social media were the two most strongly related predictors for higher adherence. Being a smoker and from a non-Mediterranean country was associated with lower adherence scores.
Zeenny et al., 2024 [46]	Adults over 18 years old (Lebanon)	Cross-sectional.	2048	LMDS	Higher adherence to the MD was associated with older age, being female, married, participating in regular physical activity, and having cardiovascular disease and diabetes. Adherence was negatively related to current and previous smokers and those with higher distress levels.
Biggi et al. 2024 [45]	Adults between the ages of 18–79 (Tunisia and Morocco)	Cross-sectional.	1617 (Morrocco: 803, Tunisia: 814)	MEDAS	Medium to low adherence was reported among Tunisian and Moroccan participants, with higher adherence observed in Morocco. Positive attitudes toward the healthiness of food were the strongest predictor of adherence, whereas picky eating was a significant negative predictor in both countries. Health motivations positively influenced adherence to the MD among Moroccans. Price and convenience were substantial barriers among Tunisians, whereas a preference for local and seasonal foods promoted adherence among Moroccans.

MEDAS: Mediterranean Diet Adherence Screener by Schroder et al. [51], MDS: Mediterranean Diet Scale by Trichoupoulo et al. [52,53], MedDietScore: a Greek Mediterranean Diet Index by Panagiotakos et al. [54], LMD: Lebanese Mediterranean diet by Naja et al. [49], LMDS: Lebanese Mediterranean Diet Scale by Zeenny et al. [46], aMed: Alternate Mediterranean Diet by Fung et al. [55]; Med-DQI: Mediterranean Diet Quality Index by Gerber et al. [56], IMI: Italian Mediterranean Index by Agnoli et al. [57], rMed score: Relative Mediterranean diet score by Buckland et al. [58], MMD: Modified Mediterranean Dietary score by Kinany et al. [42]. * We reported the data on the total general population that were presented in the included studies.

**Table 3:** Overview of the Mediterranean diet score systems that were used in the included studies.

Tool (Theoretical Range)	Used by	Utilized Score Cut-Offs		
		Low	Moderate	High
MEDAS (0–13)	[43,44]	0–5	6–7	8–13
	[45]	0–5	6–9	More than 10
aMed (0–9)	[48]	0–2.9	3–5.9	6–9
rMed score (0–18)	[48,49]	0–6.9	7–11.9	12–18
Med-DQI (0–14)	[48,49]	0–4.6	4.7–9.3	9.4–14
LMD (9–27)	[48,49]	9–14.9	15–20.9	21–27
MMD (0–12)	[42]	0–6	-	7–12
IMI (0–11)	[49]	0–3.6	3.7–7.3	7.4–11
MedDietScore (0–55)	[49]	0–18.3	18.4–36.6	36.7–55
	[47]	Not used	Not used	Not used
MDS (0–9)	[49]	0–2.9	3–5.9	6–9
Simplified MDS (0–8)	[50]	0–4	-	5–8
LMDS (0–64)	[46]	Not used	Not used	Not used

MEDAS: Mediterranean Diet Adherence Screener by Schroder et al. [51], MDS: Mediterranean Diet Scale by Trichoupoulo et al. [52,53], MedDietScore: a Greek Mediterranean Diet Index by Panagiotakos et al. [54], LMD: Lebanese Mediterranean diet by Naja et al. [49], LMDS: Lebanese Mediterranean Diet Scale by Zeenny et al. [46], aMed: Alternate Mediterranean Diet by Fung et al. [55], Med-DQI: Mediterranean Diet Quality Index by Gerber et al. [56] IMI: Italian Mediterranean Index by Agnoli et al. [57], rMed score: Relative Mediterranean score by Buckland et al. [58], MMD: Modified Mediterranean Dietary score by Kinany et al. [42].

**Table 4:** Adherence scores and distribution of population by categories of MD adherence in the included studies.

Authors, Year	Country (Sample Size)	MD Adherence Tool (Tool’s Origin)	Mean Score ± SD	Classification of Mean (Standardized Tertiles)	Classification of Mean (Original Study Cut-Offs)	Distribution of Adherence Categories Reported by the Study *		
						Low	Moderate	High
**Naja et al., 2019** [48]	Lebanon (2610)	LMD (Lebanon) rMED score (Spain) Med-DQI (France) aMed (US)	NR	NA	NA	LMD: 29.8%, rMED: 21.7% Med-DQI: 27.8% aMED: 32%	LMD: **57.5%** rMED: **71.5%** Med-DQI: **65%** aMED: **59.2%**	LMD: 12.7% rMED: 6.8% Med-DQI: 7.2% aMED: 8.8%
**El Kinany et al., 2021** [42]	Morrocco (1492)	MMD (Morrocco)	NR	NA	NA	21.0%	**56.0%**	23.0%
**Shatwan et al., 2021** [43]	Gulf countries (961)	MEDAS (Spain)	5.9 ± 2.03	Low (0–5)	Low (0–5)	**44.4%**	33.1%	22.4%
**Naja et al., 2015** [49]	Lebanon (2048)	MedDietScore (Greece) IMI (Italy) rMED score (Spain) Med-DQI (France) MDS (Europe) LMD (Lebanon)	**MedDietScore:** 27.23 ± 4.65 **IMI:** 3.56 ± 1.76 **rMED:** 8.27 ± 2.49 **Med-DQI:** 6.20 ± 1.81 **MDS:** 4.18 ± 1.49 **LMD:** 17.38 ± 3.40	**MedDietScore:** Moderate (18.4–36.6) **IMI:** Low (0–3.6) **rMED:** Moderate (6.1–12) **Med-DQI:** Moderate (4.7–9.3) **MDS:** Moderate (3–5.9) **LMD:** Moderate (15–20.9)	**MedDietScore:** Moderate (18.4–36.6) **IMI:** Low (0–3.6) **rMED:** Moderate (6.1–12) **Med-DQI:** Moderate (4.7–9.3) **MDS:** Moderate (3–5.9) **LMD:** Moderate (15–20.9)	NR	NR	NR
**El Rhazi et al., 2012** [50]	Morocco (2214)	A simplified score of MDS (Greece)	5.1 ± 1.2	Moderate (2.68–5.33)	High (5–8)	29.9%	NA	**70.1%**
**Elmskini et al., 2024** [47]	Morocco (1776)	MedDietScore (Greece)	23.27 ± 5.47	Moderate (18.4–36.6)	No categorization implemented	NR	NR	NR
**Hashim et al., 2024** [44]	UAE (1314)	MEDAS (Spain)	5.96 ± 1.92	Moderate (4.34–8.66)	Low (0–5)	36.0%	**41.0%**	23.0%
**Zeenny et al., 2024** [46]	Lebanon (2048 [Females: 1054; Males: 994])	LMDS (Lebanon)	Females: 30.90 ± 4.59 Males: 30.17 ± 4.84	Moderate for both (42.7–64)	No categorization implemented	NR	NR	NR
**Biggi et al., 2024** [45]	Morocco (803) Tunisia (814)	MEDAS (Spain)	Morrocco: 7.62 ± 1.84; Tunisia: 7.21 ± 1.91	Moderate for both (4.34–8.66)	Moderate for both (6–9)	Morocco: 11.8%, Tunisia: 19.4%	Morocco: **73.6%**, Tunisia: **69.3%**	Morocco: 14.6%, Tunisia: 11.3%

MEDAS: Mediterranean Diet Adherence Screener by Schroder et al. [51], MDS: Mediterranean Diet Scale by Trichoupoulo et al. [52,53], MedDietScore: a Greek Mediterranean Diet Index by Panagiotakos et al. [54], LMD: Lebanese Mediterranean diet by Naja et al. [49], LMDS: Lebanese Mediterranean Diet Scale by Zeenny et al. [46], aMed: Alternate Mediterranean Diet by Fung et al. [55], Med-DQI: Mediterranean Diet Quality Index by Gerber et al. [56], IMI: Italian Mediterranean Index by Agnoli et al. [57], rMed score: Relative Mediterranean score by Buckland et al. [58], MMD: Modified Mediterranean Dietary score by Kinany et al. [42]. NA: Not applicable; NR: not reported. * The categories with the highest percentage are highlighted in bold.

## Data Availability

All the datasets used and/or analyzed during the current study are available from the corresponding author upon reasonable request.

## Supplementary Materials

The following supporting information can be downloaded at: https://www.mdpi.com/article/10.3390/healthcare13172217/s1, Table S1: Electronic databases used with relevant search periods and terms. Table S2: Quality Assessment by JBI Critical Assessment Tool of Analytical Cross-sectional Studies [41].
