# Supplementary material for: Adherence to the Mediterranean Diet Across the League of Arab States: A Systematic Review
Source: Healthcare (Basel). 2025 Sep 4;13(17):2217. doi: 10.3390/healthcare13172217 (PMC12427626; doi:10.3390/healthcare13172217)
Supplement: Supplementary file 1 [file healthcare-13-02217-s001.zip › healthcare-3723262-supplementary.pdf]

Supplementary Table S1. Electronic databases used with relevant search periods and terms

Databases	Search period	MeSH keywords, terms, phrases, and Boolean operators
Cochrane Database; ArticleFirst; Biomed Central; BioOne; BIOSIS; CINAHL; EBSCOHost; JSTOR; ProQuest; PubMed; SAGE Reference Online; ScienceDirect; Scopus; SpringerLink; Taylor & Francis; Wiley Online	2010 and including November 2024	Mediterranean diet OR Mediterranean dietary pattern [MeSH Terms]<br><br>AND<br><br>Adherence [MeSH Terms]<br><br>AND<br><br>Algeria OR Egypt, OR Bahrain, OR Comoros, OR Djibouti, OR Iraq, OR Jordan, OR Saudi Arabia, OR Kuwait, OR Lebanon, OR Libya, OR Mauritania, OR Morocco, OR Oman, OR Palestinian Territories, OR Qatar, OR Yemen, OR Somalia, OR Sudan, OR Syria, OR Tunisia, OR United Arab Emirates, OR Arab League, OR Arab League States [MeSH Terms]

Supplementary Table S2. Quality Assessment by JBI Critical Assessment Tool of Analytical Cross-sectional Studies [1]

Study	Q1. Were the criteria for inclusion in the sample clearly stated? Defined?	Q2. Were the study subjects and the setting described in Detail?	Q3. Was the exposure measured in a valid and reliable Way?	Q4. Were objective, standard criteria used for measurement of the condition?	Q5. Were confounding factors identified?	Q6. Were there strategies to deal with confounding factors Stated?	Q7. Were the outcomes measured in a valid and reliable Way?	Q8. Was an appropriate statistical analysis used?	Risk Of Bias Score (%)	Risk of Bias
Naja et al., 2019 [2] [a]	Yes	Yes	Yes	NA	Yes	Yes	Yes	Yes	7/7 (100%)	Low
El Kinany et al., 2021 [3] [a]	Yes	Yes	Unclear	Yes	Yes	Yes	Yes	Yes	7/8 (87.5%)	Low
Elmskini et al., 2024 [4] [a]	Yes	Yes	Yes	Yes	Unclear	No	Yes	Unclear	5/8 (62.5%)	Moderate
Shatwan et al., 2021 [5] [a]	Yes	Yes	Yes	Yes	Yes	Yes	Yes	Yes	8/8 (100%)	Low
Zeenny et al., 2024 [6] [a]	Yes	Yes	Unclear	Yes	Yes	Yes	Unclear	No	5/8 (62.5%)	Moderate
Biggi et al. 2024 [7] [b]	Yes	Yes	Yes	Yes	Unclear	Unclear	Yes	Yes	6/8 (75%)	Low
Naja et al., 2015 [8] [b]	Yes	Yes	Yes	Yes	Yes	Yes	Yes	Unclear	6/8 (75%)	Low
El Rhazi et al., 2012 [9] [b]	Yes	Yes	Yes	Yes	Yes	Yes	Yes	No	6/8 (75%)	Low
Hashim et al., 2024 [10] [b]	Yes	Yes	Yes	Yes	Yes	Yes	Yes	No	6/8 (75%)	Low

[a] Studies that had MD adherence assessed as the exposure variables, and other outcomes as the condition variable.
[b] Studies that had sociodemographic/lifestyle variables assessed as the exposure variables, and the MD adherence as the condition variable.
Each item in the assessment was assigned one of four possible answers: **Yes**, **No**, **Unclear**, or **Not Applicable**. Responses of *Yes* were scored as **1**, while *No* and *Unclear* were scored as **0**. If an item was not applicable, the question was skipped and excluded from the percentage calculation. The **percentage of applicable items scored as 'Yes'** was then used to determine the **risk of bias**:

- **High risk** if ≤49% of items were scored as 'Yes'
- **Moderate risk** if 50%–69% were scored as 'Yes'
- **Low risk** if >70% were scored as 'Yes'

1. Barker, T.H., et al., *The revised JBI critical appraisal tool for the assessment of risk of bias for analytical cross-sectional studies.* JBI Evidence Synthesis, 2025: p. 441-453.
2. Naja, F., et al., *Mediterranean diet and its environmental footprints amid nutrition transition: the case of Lebanon.* Sustainability, 2019. **11**(23): p. 6690.
3. El Kinany, K., et al., *Modified Mediterranean diet score adapted to a southern Mediterranean population and its relation to overweight and obesity risk.* Public Health Nutrition, 2021. **24**(13): p. 4064-4070.
4. Elmskini, F.Z., et al., *Increased nutrition knowledge and adherence to the Mediterranean diet are associated with lower body mass index and better self-rated general health among university students.* Human Nutrition & Metabolism, 2024. **35**: p. 200240.
5. Shatwan, I.M., et al., *High Adherence to the Mediterranean Diet Is Associated with a Reduced Risk of Obesity among Adults in Gulf Countries.* Nutrients, 2021. **13**(3).
6. Zeenny, R.M., et al., *Adherence to the Mediterranean Diet and Cardiovascular Risk Factors among the Lebanese Population: A Nationwide Cross-Sectional Post Hoc Study.* Nutrients, 2024. **16**(15): p. 2426.
7. Biggi, C., et al., *Drivers and Barriers Influencing Adherence to the Mediterranean Diet: A Comparative Study across Five Countries.* Nutrients, 2024. **16**(15): p. 2405.
8. Naja, F., et al., *A novel Mediterranean diet index from Lebanon: comparison with Europe.* European journal of nutrition, 2015. **54**: p. 1229-1243.
9. El Rhazi, K., et al., *Adherence to a Mediterranean diet in Morocco and its correlates: cross-sectional analysis of a sample of the adult Moroccan population.* BMC Public Health, 2012. **12**: p. 1-8.
10. Hashim, M., et al., *Determinants for Mediterranean diet adherence beyond the boundaries: a cross-sectional study from Sharjah, the United Arab Emirates.* Journal of Translational Medicine, 2024. **22**(1): p. 513.
